# **­­­**A web resource for nutrient use efficiency-related genes, quantitative trait loci and microRNAs in important cereals and model plants

**DOI:** 10.12688/f1000research.14561.1

**Published:** 2018-05-29

**Authors:** Anuj Kumar, Ajay Pandeya, Girik Malik, Mansi Sharma, Hima Kumari P., Anil Kumar S., Vijay Gahlaut, M.N.V. Prasad Gajula, Krishna Pal Singh, Prashanth Suravajhala, Harindra Singh Balyan, Pushpendra K. Gupta

**Affiliations:** 1 Advanced Centre for Computational and Applied Biotechnology, Uttarakhand Council for Biotechnology (UCB), Dehradun, Uttarakhand, 248007, India; 2 Department of Biotechnology, Graphic Era University, Dehradun, Uttarakhand, 248002, India; 3 Bioclues.org, Hyderabad, 500072, India; 4 Labrynthe, New Delhi, India; 5 Bioinformatics Laboratory, Institute of Cytology and Preventative Oncology, Noida, 201301, India; 6 Department of Genetics, Osmania University, Hyderabad, Telengana, 500007, India; 7 Department of Genetics and Plant Breeding, Chaudhary Charan Singh University, Meerut, Uttar Pradesh, 250004, India; 8 Institute of Biotechnology, Professor Jayashankar Telangana State Agricultural University, Rajendranagar , Telangana, 500030, India; 9 Department of Biotechnology and Bioinformatics, Birla Institute of Technology & Science, Jaipur, Rajasthan, 302001, India

**Keywords:** Nutrient use efficiency, cereals, web-resource, functional genomics, genes, QTLs, miRNAs, stress

## Abstract

Cereals are key contributors to global food security. Genes involved in the uptake (transport), assimilation and utilization of macro- and micronutrients are responsible for the presence of these nutrients in grain and straw. Although many genomic databases for cereals are available, there is currently no cohesive web resource of manually curated nutrient use efficiency (NtUE)-related genes and quantitative trait loci (QTLs). In this study, we present a web-resource containing information on NtUE-related genes/QTLs and the corresponding available microRNAs for some of these genes in four major cereal crops (wheat ( *Triticum aestivum*), rice ( *Oryza sativa*), maize ( *Zea mays*), barley ( *Hordeum vulgare*)), two alien species related to wheat ( *Triticum urartu* and *Aegilops tauschii*), and two model species ( *Brachypodium distachyon *and *Arabidopsis thaliana*). Gene annotations integrated in the current web resource were manually curated from the existing databases and the available literature. The primary goal of developing this web resource is to provide descriptions of the NtUE-related genes and their functional annotation. MicroRNAs targeting some of the NtUE related genes and the QTLs for NtUE-related traits are also included. The genomic information embedded in the web resource should help users to search for the desired information.

## Introduction

The world population is expected to be >9.6 billion by 2050. As a consequence, food production must increase by 70% to meet the growing demand for food; this translates into 3 billion tons of additional cereal grain ^1^. In view of this, food and nutritional security have been central to several international discussions, involving development of strategies for enhanced and sustainable food production ^2^. These strategies include the development of cultivars that would be (i) resilient to climate change, (ii) tolerant to biotic and abiotic stresses and (iii) efficient to the use of fertilizer and water resources. In connection with strategy (iii), it is well known that the present day high-yielding cultivars of a majority of crops have a high demand for inputs including water and nutrients.

Out of the 17 essential elements needed for plant growth and development, three non-mineral elements [C (carbon), H (hydrogen) and O (oxygen)] are derived by the plants from CO _*2*_ and H _2_O, and the remaining 14 mineral elements are ordinarily derived from the soil as inorganic salts. These mineral elements include: (i) six macronutrients [N (nitrogen), P (phosphorous), K (potassium), S (sulphur), Ca (calcium) and Mg (magnesium)] that are required in large quantities (1,000–15,000 mg/kg of plant dry weight); and (ii) eight micronutrients [B (boron), Cl (chlorine), Cu (copper), Fe (iron), Mn (manganese), Mb (molybdenum), Zn (zinc) and Ni (nickel)] that are needed in relatively small quantities (0.1–100 mg/kg of plant dry weight) ^3^.

Since most soils are deficient in N, P and K, these elements are applied to the crops in the form of chemical fertilizers to help their proper growth and development, eventually leading to improved grain yield ^4, 5^. Of these three macronutrients, N and P are required in the maximum quantities by the plants ^6^. With the increased future demand for cereal grains, especially within developing countries, the demand for N and P is likely to grow ^7^.

During the last four decades, the doubling of food production worldwide has been associated with 7-fold increase in the consumption of N fertilizers and 3.5-fold increases in the consumption of P fertilizers ^8, 9^. It is estimated that a further doubling of global food production during the next three decades would require a 3.15-fold increase in total N application and 2.5-fold increase in total P application ^8^. The enhanced use of chemical fertilizers would also add up to 50% (for N fertilizers) of the operational costs in agriculture ^4^.

It is estimated that 30–50% of N applied in the fields is used by the crops ^10^, with the excess N and ammonia converted by the soil bacteria into nitrate and nitrite, which are lost via ** emission in the form of NH _3_, N _2_O and NO _2_ gases, and/or leached out into the soil in the form of nitrate ions (NO _3_^−^) ^4, 11^, causing eutrophication in terrestrial and aquatic systems. N _2_O is 300 times more potent as a greenhouse gas (GHG) than CO _2_; 70% of N _2_O is contributed by natural sources and is responsible for considerable GHG effects, which have a significant influence on climate change ^7, 12, 13^.

 Unlike N, part of the P derived from applied chemical fertilizers is held very tightly by the surface of the soil particles or is fixed as organic P compounds, thus remaining unavailable to the plant for uptake; P is also lost through leaching into the ground and surface waters, damaging the surrounding environment ^14^. The leached P also leads to eutrophication and associated algal blooms, decreased dissolved O _2_, foul odour, and generally poor water quality ^15^. To further exacerbate the problem, the P used in fertilizers is obtained from rock phosphate, which is a non-renewable source and should be depleted in the next 50–100 years ^16^. Indirectly through the food chain, application of enhanced N and P fertilizer also leads to widespread deficiency of micronutrients in human population, thus making it a health hazard.

In view of the importance of nutrient use efficiency (NtUE) as an agronomic practice in crop breeding, the European Commission recently concluded a 5-year research project 'NUE-CROPS' with the aim of improving NtUE in four major European food, feed and biofuel crops to reduce the negative environmental impact of crop production ( EU-FP7 222-645). The findings of this project were discussed at a EUCARPIA section meeting on 'Organic and Low-input Agriculture' during September 24–26, 2013 at the George August University of Gottingen and the proceedings were published in Euphytica ^17^.

Traditional breeding practices involving higher application of chemical fertilizers are no longer providing yield improvements ^18^. The Food and Agriculture Organization of the UN has noticed that since the mid-1980s, crops, including wheat, maize and soybean, have shown only 1% annual growth in their productivity, whereas in developed countries the annual growth in crop yield has remained static, with no improvement in productivity ^19^. Thus it has been a challenge for the geneticists and plant breeders to achieve the desired increase in the productivity of cereal crops in particular, and that of the other food crops in general, without compromising the quality of food and environment health. Of the suggested strategies, the development of NtUE crop plants through genetic intervention is the best approach, which is also environment-friendly. This approach would require reduced levels of fertilizer application, with the desired increase in current grain yield levekls ^7, 9, 14, 19^. Also termed "An Evergreen Revolution" and the “Second Green Revolution”, this approach would allow increased productivity, while following sustainable agricultural practices ^20, 21^.

 An increased understanding of the NtUE at the genetic and molecular levels would certainly help in developing NtUE cultivars giving enhanced crop productivity. The NtUE (ratio of grain yield to units of nutrient supplied or available) can be divided in two components: (i) nutrient uptake efficiency (the ratio of total plant nutrients to the available or supplied nutrient), which is the ability of the crop plant to extract nutrients from the soil; and (ii) nutrient utilization efficiency (the ratio of grain yield to total plant nutrients in grain and straw), which measures the capacity of the plant to convert the absorbed nutrient into grain yield ^22, 23^. Another approach is to calculate the above-ground biomass NtUE, which is measured by dividing the dry shoot weight by the nutrient supplied ^24^; however, plant breeders are most often interested in the first approach of measuring the NtUE in breeding of grain crops.

 The NtUE is a complex trait, involving signaling, acquisition, transport and utilization, the latter constituting assimilation and translocation/remobilization ^25^. The detailed description of the mechanisms of signaling, uptake, transport and utilization of the nutrients is neither necessary nor desirable in this article, but we know that they form an interwoven network of genetic control. Thus, the identification of genes/QTLs and the microRNAs (miRNAs) targeting the NtUE-related genes involved in the uptake, transport and utilization of nutrients in different parts of the plant are important targets for breeding cultivars with improved NtUE. The genes that have been identified or are likely to be identified in future, or the DNA markers associated with these genes/QTLs as well as the information related to miRNA can be exploited as follows: (i) marker-assisted selection aimed at the selection of desirable genotypes exhibiting improved plant performance under reduced nutrient supply; (ii) a study of allelic variation of the genes/QTLs for their subsequent exploitation in plant breeding, and (iii) the development of transgenics with improved NtUE ^25, 26^.

A number of genomic resources are freely available for cereals and include the following: Gramene ^27^, PIGD ^28^, PlantTFDB 43.0 ^29^, PSPDB ^30^, CerealsDB ^31^, MetaCrop 2.0 ^32^, Phytozome ^33^, PlantGDB ^34^, CSR-DB ^35^, CR-EST ^36^ and GrainGenes ^37^. However, a similar web resource for NtUE genes or QTLs is not available, although the USDA has developed a tool for calculating the approximate amounts of nutrients (N, P and K) required for an optimum harvest of each agricultural crop. Therefore, we have developed the NtUE web-resource, which provides comprehensive information on genes related to the components of NtUE that are involved in processes such as the uptake, transport, nitrate assimilation, re-assimilation, amino acid biosynthesis, C/N storage and metabolism, signaling and regulation, translocation, remobilization, senescence, regulation, DNA-binding, ion-binding and copper homeostasis. The web resource also contains information on QTLs for different NtUE-related traits, such as shoot growth, total N, nitrate, free-amino acid contents, relative grain yield, relative biomass yield, relative grain N, relative biomass N, N response, leaf length, P starvation, and Fe and Zn homeostasis. The miRNAs targeting some of the NtUE-related genes are also included in the web resource. The NtUE web resource is, to our knowledge, the first of its kind. We believe that the web resource will be useful for members of the plant research community, especially plant breeders, who may like to plan experiments related to the improvement of NtUE of cereal crop plants.

## Methods

### Collection of NtUE-responsive genes

We populated the entries in the web resource by retrieving NtUE-related genes using gene ontology keyword searches for nutrients, plants, crops and their NtUE-related role from GenBank, EnsemblPlants, Gramene and UniProt ^38^. We also extensively searched PubMed with different keywords or their combination to find relevant articles.

### Annotation of NtUE genes

We performed functional annotation studies at gene and protein level to develop comprehensive information for the collected NtUE responsive genes. Complementary- and target-site accessibility both are important factors of plant regulatory small RNA target recognition mechanism ^39^. To predict potential miRNAs targeting the NtUE responsive genes in plant species, the web-based psRNATarget server ^40^ was used with the default parameters. Further the QTLs for NtUE related traits were manually curated from the available scientific literatures, GrainGenes database and MaizeGDB.

### Database construction, implementation and operation

**Figure 1.**
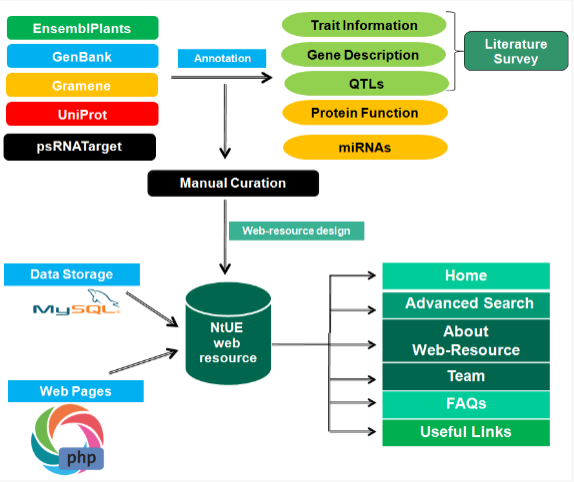
Flow chart depicting the steps involved in preparation of the nutrient use efficiency (NtUE) web resource and acquisition of the stored data.

The NtUE web resource was set up based on three-tier architecture concept using Apache/PHP/MySQL on a Windows 8.1 platform. An integrated system driven through MySQL (5.6.21) and PHP (5.6.24) was developed to handle the storage of annotated 688 genes in the web resource. The database can be accessed from any operating system client recommended with Java and internet connection. A flow-chart depicting the steps involved in preparation of web resource and acquisition of data by the user is presented in Figure 1. All figures have been drawn using MS office tools.

## Use of NtUE web resource

### Overview

NtUE is a comprehensive web resource containing information on NtUE-related genes/QTLs and the microRNAs targeting some of these genes in three major cereal crops (wheat ( *T. aestivum*), rice ( *O. sativa*), maize ( *Z. mays*)), two alien species ( *T. urartu* and *A. tauschii*) related to wheat, and two model species ( *B. distachyon* and *A. thaliana*).

In the current release, we have compiled 688 NtUE-responsive gene records that were manually curated and annotated using different bioinformatics-based methods. Each entry embedded in this web resource has been arranged in a gene-centric manner for easy access and retrieval.

### Gene-centric portal

**Figure 2.**
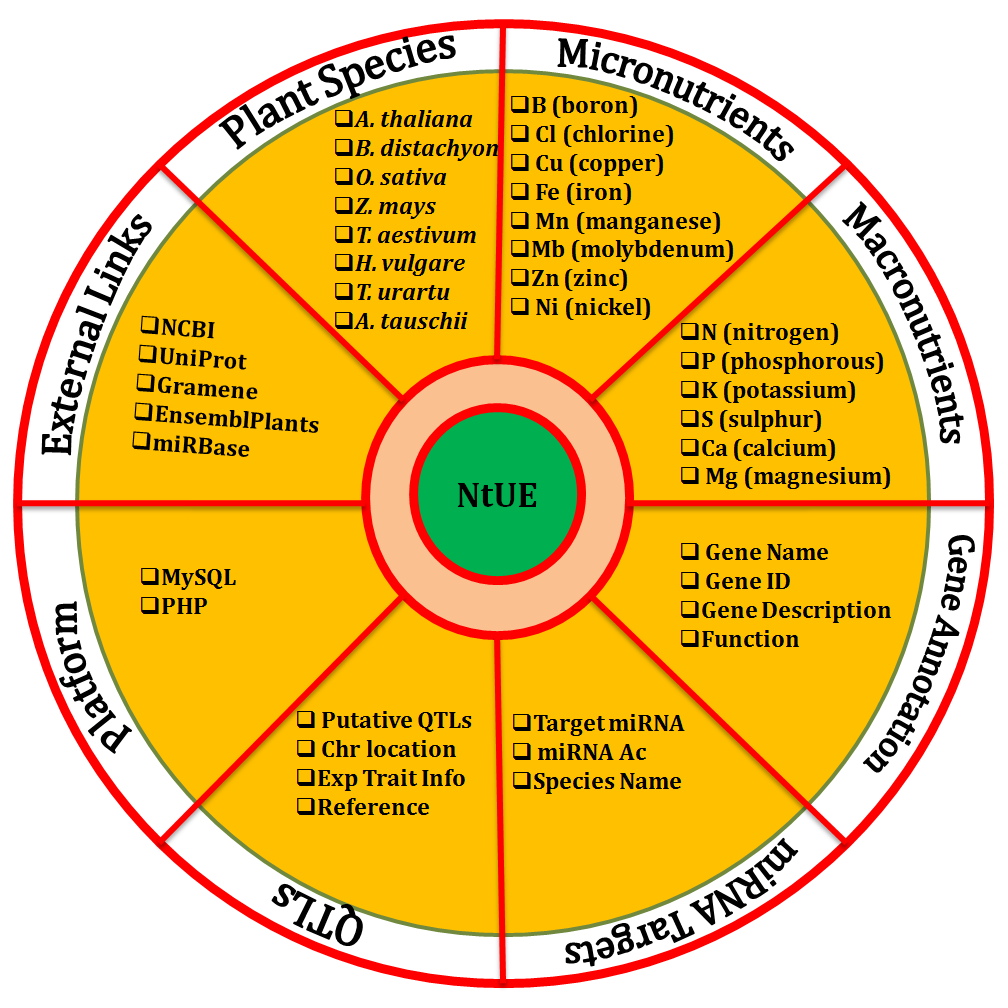
Schematic showing information available in the nutrient use efficiency (NtUE) web resource.

Each entry embedded in the web resource provides the following comprehensive information on NtUE-responsive genes: (i) organism name; (ii) type of associated nutrient (macronutrients and micronutrients); (iii) gene symbol; (iv) gene ID (entry accession on UniProt, GenBank, Gramene and EnsemblPlants), with an active hyperlink that provides genomic information and sequences of entry on portal of parental database; (v) gene description; (vi) function; (vii) linked miRNAs with an active hyperlink to miRBase database; (viii) putative QTLs; (ix) chromosomal location marker; (x) information on experimental condition of the trait; and (xi) references for QTLs (as shown in Figure 2).

### Data access

**Figure 3.**
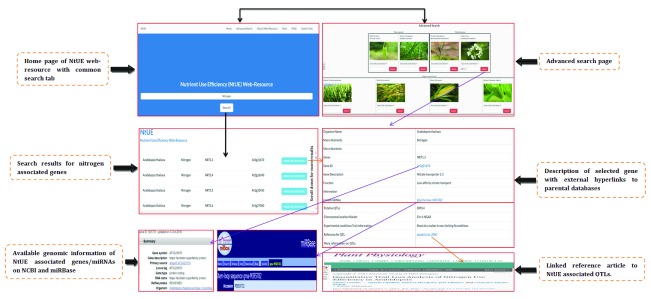
An illustration of the home page, search options, search summary and search results along with details of genomic information of nutrient use efficiency (NtUE)-associated genes on NCBI, information on the miRBase database regarding targeted miRNAs and a linked reference article to quantitative trait loci (QTLs) on journal homepage.

The information available in the web resource could be accessed through keyword search tab by assigning the nutrient name (e.g. N, P, K, Zn, Fe), organism name (e.g. *Arabidopsis thaliana*, *Brachypodium distachyon*, *Oryza sativa*, *Triticum aestivum*, *Hordeum vulgare*), gene name (NRT1.1, PHT1.1), accession number and clicking on the “search” button. After submitting the keyword, a page will open presenting a list of NtUE-responsive gene details, which contains the specific function of the gene. Clicking on “MORE INFORMATION” will direct the user to entire information for a particular gene. This scroll down based page provides comprehensive information that includes a summary of gene including: organism name, type of nutrient (macronutrients and micronutrients), gene information (gene symbol, gene ID, gene description, function) target miRNAs, and QTLs (chromosomal location marker, information on experimental condition of the trait and references for QTLs). Alternatively, the user can search the NtUE web resource using the advanced search option, which will allow them to conduct an organism-specific search using a gene name (e.g. NRT1.1, PHT1.1) or accession number. Using a specific example of search option, the steps involved in using the keyword-based search and advanced search options are shown in Figure 3. The NtUE web resource has a user-friendly entry point for each gene/miRNA/QTL. Each gene with a corresponding miRNA and the record of the corresponding QTL reference article in the web resource is linked to external resources, including NCBI, UniProt, Gramene, EnsemblPlants and miRBase. Using external links, users can get detailed genomic and proteomic information on NtUE-associated genes, miRNA-target genes and the trait for each QTL. Additionally, it was ensured that throughout, standard terminology is used, validated and processed, making the tool an easily understandable tool for accessing genomic information using NtUE. QTLs embedded in the NtUE web resource contain the chromosomal location, marker interval, trait information and reference article, with an external link to the journal in which the article is published. Figure 3 shows an example of how more information on genes and targeted miRNAs can be retrieved.

### Results of data analysis

**Figure 4.**
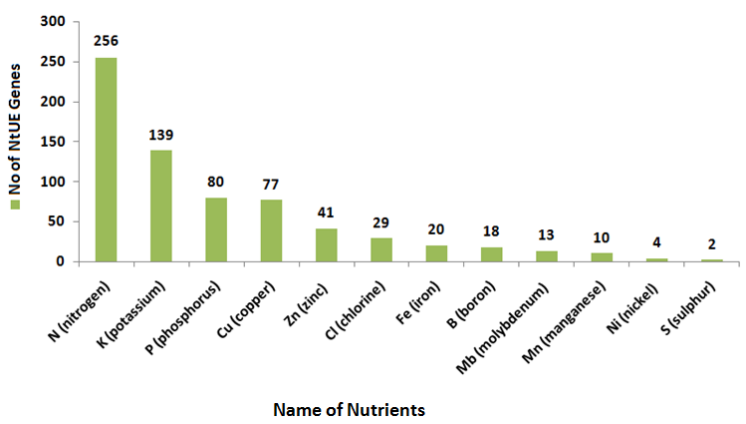
Distribution of macronutrient- and micronutrient-responsive genes corresponding to their nutrient type in the nutrient use efficiency (NtUE) web resource.

***Type of nutrients***. At present, the NtUE web resource covers 668 NtUE-responsive genes, corresponding to 12 nutrient types (the nutrient-wise distribution of NtUE genes in the NtUE web resource is shown in Figure 4). It is apparent from the graph that the majority of NtUE genes are associated with N. The other major nutrients embedded in the web resource for which NtUE genes have been detected are K, P, Cu and Zn.

**Figure 5.**
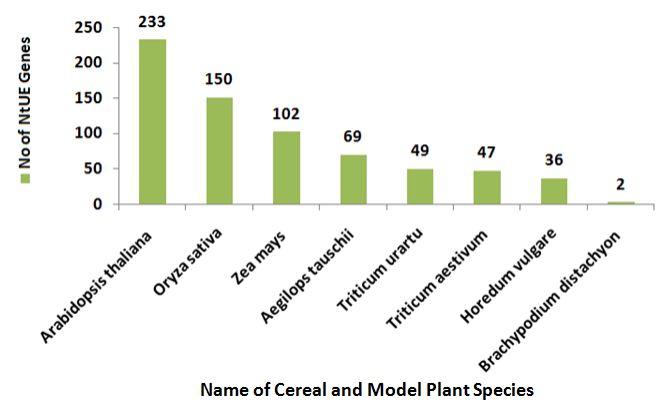
Distribution of macronutrient- and micronutrient-responsive genes in cereal and model plant species embedded in the nutrient use efficiency (NtUE) web resource.

***NtUE-responsive genes in cereals and model plant species***. NtUE-responsive genes have been reported in literature published in peer-reviewed journals. These genes have been detected from the genomes of eight model and cereal plant species (embedded in the web resource), including Arabidopsis, rice, wheat and maize ( Figure 5). Among all the embedded plant genomes, Arabidopsis are the best-explored source of annotated NtUE-responsive genes in the NtUE web resource.

***Comparison of NtUE web resource with Gramene***. Gramene is a biological repository that provides genomic information about members of the Poaceae family. Gramene is also an experimentally based comprehensive database consisting of genomic sequences, chromosomal locations, associated QTLs and markers for genes from various popular genomes belonging to the Poaceae family. In contrast, the NtUE web resource is a literature-curated resource for NtUE-responsive genes of model-organisms and cereals; these genes differ for different nutrients and in different plant genomes. The NtUE web resource not only lists the NtUE genes according to their nutrient type, but also provides data on aspects such as miRNA targets, associated QTLs, marker associated with QTL, information on trait and experiment and external links to literature on QTL. Only NtUE genes from cereals overlap with those in Gramene, but the NtUE genes from model plant species are unique to the NtUE web resource.

## Conclusions

The NtUE web resource is, to our knowledge, the first of its kind to provide a comprehensive non-redundant catalogue of NtUE genes that have a vital role in model and cereal plant species during plant growth and development, with supporting evidence from published literature. We hope that this web resource will be a useful compendium to the geneticists, plant breeders and computational biologists involved in crop improvement. The resource contains manually curated entries with an adequate level of stringency to select only those genes/QTLs/miRNAs that are validated and linked to NtUE. This resource will help in providing new solutions to plant geneticists and breeders who are aiming towards the sustainable use of limited soil nutrients for enhanced productivity without compromising the nutritional quality of the grains. Information on more than half of these genes, QTLs and miRNAs is not available in the previously generated databases. With further progress in the identification of genes/QTLs/miRNAs for NtUE related traits, we anticipate that these numbers will eventually grow by two- to three-fold.

## Data and software availability

The dataset for NtUE web-resource is publically available on OMIC TOOLS with unique identifier OMICS_24069.

The archived PHP files for the web resource at the time of publication have been deposited at Zenodo: https://doi.org/10.5281/zenodo.1233011 ^41^.

License: Creative Commons Attribution 4.0.
