# Supplementary material for: ­­­A web resource for nutrient use efficiency-related genes, quantitative trait loci and microRNAs in important cereals and model plants
Source: F1000Res. 2018 May 29;7:ISCB Comm J-673. [Version 1] doi: 10.12688/f1000research.14561.1 (PMC6073097; doi:10.12688/f1000research.14561.1)
Supplement: Peer review history

# Peer review: Referee response for version 1

**DOI:** 10.5256/f1000research.15848.r36463

**Published:** 2018-08-02

**Authors:** Vishal Acharya

**Affiliations:** 1 Biotechnology Division, CSIR-Institute of Himalayan Bioresource Technology, Palampur, India

The authors have well prepared the manuscript dealing with critical information about necessary for construction of database of nutrient use efficiency-related genes (NtUE) in cereals and model plants.

 The authors suggested that this is first of kind to provide comprehensive information dealing with NtUE. The authors have compared also with the known database Gramene which is widely used and suggested the advantage in providing more information of model plants apart from cereals where Gramene only stand. 

 The overall conclusion is the paper is well researched and needs to be indexed.

I have read this submission. I believe that I have an appropriate level of expertise to confirm that it is of an acceptable scientific standard.

# Peer review: Referee response for version 1

**DOI:** 10.5256/f1000research.15848.r34482

**Published:** 2018-06-18

**Authors:** Rajib Bandopadhyay

**Affiliations:** 1 UGC-CAS, Department of Botany, The University of Burdwan, Burdwan, India

1. Resolution of the figures are poor and better resolution figures must be supplemented.
2. There are some minor spelling mistakes in Page No. 3; like ‘levekls’ which should be written as ‘levels’.
3. In Figure 5, scientific name of the plant species should be written in italics.
4. In the manuscript, several grammatical mistakes are observed which can be amended in a way where the entire manuscript can be written in a same grammatical manner.

 

 Overall the NtUE web source is a good comprehensive catalogue on model cereal plant species, their growth and development and the manuscript has good scientific outcome.

I have read this submission. I believe that I have an appropriate level of expertise to confirm that it is of an acceptable scientific standard.
